# A Simple Detection Method for Low-Affinity Membrane Protein Interactions by Baculoviral Display

**DOI:** 10.1371/journal.pone.0004024

**Published:** 2008-12-24

**Authors:** Toshiko Sakihama, Takato Sato, Hiroko Iwanari, Toshio Kitamura, Shimon Sakaguchi, Tatsuhiko Kodama, Takao Hamakubo

**Affiliations:** 1 Department of Molecular Biology and Medicine, The University of Tokyo, Tokyo, Japan; 2 Laboratory for Systems Biology and Medicine, Research Center for Advanced Science and Technology, The University of Tokyo, Tokyo, Japan; 3 Division of Cellular Therapy, Institute of Medical Science, The University of Tokyo, Tokyo, Japan; 4 Department of Experimental Pathology, Institute for Frontier Medical Sciences, Kyoto University, Kyoto, Japan; University Paris Sud, France

## Abstract

**Background:**

Membrane protein interactions play an important role in cell-to-cell recognition in various biological activities such as in the immune or neural system. Nevertheless, there has remained the major obstacle of expression of the membrane proteins in their active form. Recently, we and other investigators found that functional membrane proteins express on baculovirus particles (budded virus, BV). In this study, we applied this BV display system to detect interaction between membrane proteins important for cell-to-cell interaction in immune system.

**Methodology/Principal Findings:**

We infected Sf9 cells with recombinant baculovirus encoding the T cell membrane protein CD2 or its ligand CD58 and recovered the BV. We detected specific interaction between CD2-displaying BV and CD58-displaying BV by an enzyme-linked immunosorbent assay (ELISA). Using this system, we also detected specific interaction between two other membrane receptor-ligand pairs, CD40-CD40 ligand (CD40L), and glucocorticoid-induced TNFR family-related protein (GITR)-GITR ligand (GITRL). Furthermore, we observed specific binding of BV displaying CD58, CD40L, or GITRL to cells naturally expressing their respective receptors by flowcytometric analysis using anti-baculoviral gp64 antibody. Finally we isolated CD2 cDNA from a cDNA expression library by magnetic separation using CD58-displayng BV and anti-gp64 antibody.

**Conclusions:**

We found the BV display system worked effectively in the detection of the interaction of membrane proteins. Since various membrane proteins and their oligomeric complexes can be displayed on BV in the native form, this BV display system should prove highly useful in the search for natural ligands or to develop screening systems for therapeutic antibodies and/or compounds.

## Introduction

Cell surface proteins mediate intercellular recognition and signal transduction. In the immune system, the interaction of an antigen-specific T cell with an antigen presenting cell (APC) such as a B cell or a dendritic cell (DC) is mediated by the engagement of a diverse array of receptors on one cell, with ligands (or counter receptors) on the opposing cell, both of which are membrane proteins [1]. Such interactions between membrane proteins lead to various immune responses such as cytokine secretion, antibody production or the killing of target cells. In many cases, however, it has been difficult to detect these receptor-ligand interactions by conventional techniques, because the affinity of interaction between these membrane proteins is generally low (Kd∼1 µM) [2]. It is likely that oligomerization of these proteins on the cell surface increases the avidity and stabilizes the interaction. Since it is difficult to reconstitute such oligomerization using the soluble monomeric form of these transmembrane proteins, several different systems have been attempted. One is the fusion of extracellular domain of membrane proteins and the Fc portion of immunoglobulin [3], [4]. Another is the engraftment of chelator-lipid liposomes with the C-terminal hexahistidine-tagged extracellular domain of membrane proteins [5]. However, some membrane proteins may not retain the proper conformations required to bind ligand (or receptor) after such manipulations.

To detect membrane protein interaction, heterotypic cell adhesion assay has also been utilized. In this assay, two different types of cells individually expressing receptors or ligands on their surfaces are mixed, and heterotypic binding cells are detected either by microscopy or by labeling with a radioisotope or fluorochrome [6]–[8]. While this system allows detection of the interaction of native transmembrane proteins and their oligomers, endogenous receptor or other proteins may affect assays.

Meanwhile, there is accumulating evidence that heterologous membrane proteins are displayed on the extracellular baculovirus particles (budded virus, BV) (reviewed in [9]). We and other investigators have reported that membrane proteins such as cell surface receptors [10]–[12], a transporter [13], or enzymes [12], [14] express on BV in biologically active form. Furthermore, the co-expression of multiple proteins results in the formation of functional protein complex on BV, as shown by the complex between G protein-coupled receptor and heterotrimeric G protein subunits [11] as well as the four-protein complex of γ-secretase [14]. Since Sf9 insect cells, the host of baculovirus, are essentially free of the homologues of mammalian immune receptors or their ligands, this BV display system holds promise for its ability to provide a low background environment which would enable detection of the interaction between receptors and ligands.

In this study, we attempted to further utilize this BV display method to develop a system for detecting interactions between receptors and ligands, both of which are membrane proteins.

## Results

### Detection of receptor-ligand interaction between membrane proteins displayed on BV

**Figure 1:**
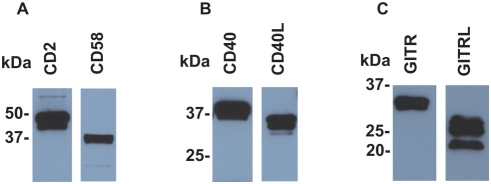
Expression of heterologous membrane proteins in BV fraction confirmed by Western blot. Ten micrograms of each of the BV samples expressing CD2 or its ligand CD58 (A), CD40 or CD40L (B), and GITR or GITRL (C) were loaded in each lane. The blotted membranes were immuno-stained with either anti-FLAG or anti-HA antibodies according to the attached tag as described in Materials and Methods. The positions of the molecular mass marker proteins are indicated on the *left*.

**Figure 2:**
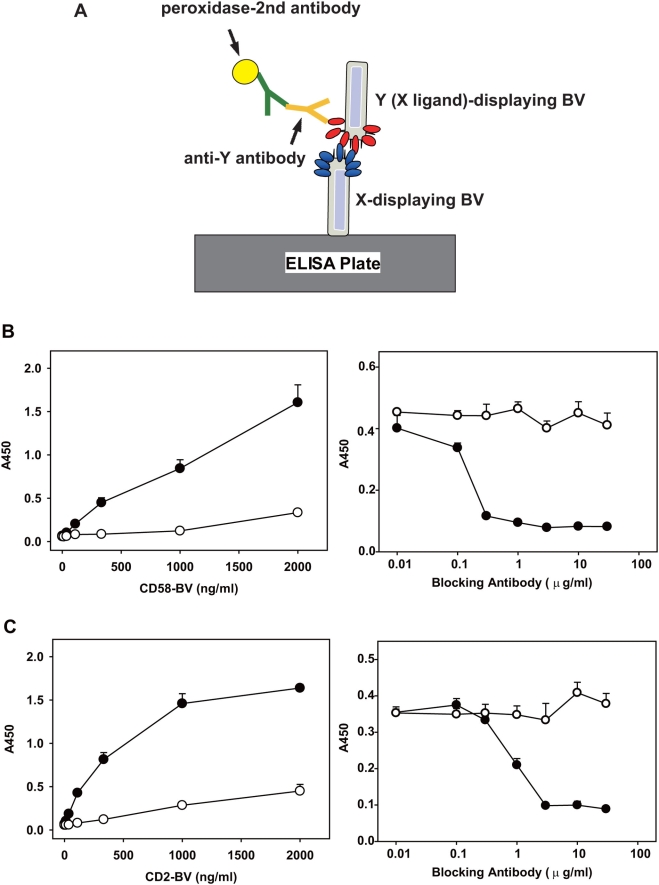
Detection of specific interaction between CD2 and CD58 individually displayed on BV. (A) Schematic view of the ELISA system. Details are described in the text. X and Y correspond to CD2 and CD58 (or vice versa for C) respectively. (B) Left panel; binding of CD58-BV to immobilized CD2-BV (filled circles) or wild-type BV (open circles). Right panel: Blocking of CD58-BV binding to the plate-bound CD2-BV by pre-incubation of wells with an anti-CD2 antibody (filled circles) or control mouse IgG1 (open circles). (C) Left panel: binding of CD2-BV to the immobilized CD58-BV (filled circles) or wild-type BV (open circles). Right panel: blocking of CD2-BV binding of the plate-bound CD58-BV by pre-incubation of wells with an anti-CD58 antibody (filled circles) or control mouse IgG2a (open circles). Each well was coated with 0.5 µg of BV. Each data point represents detection in triplicate (error bar, 1 s.d.).

**Figure 3:**
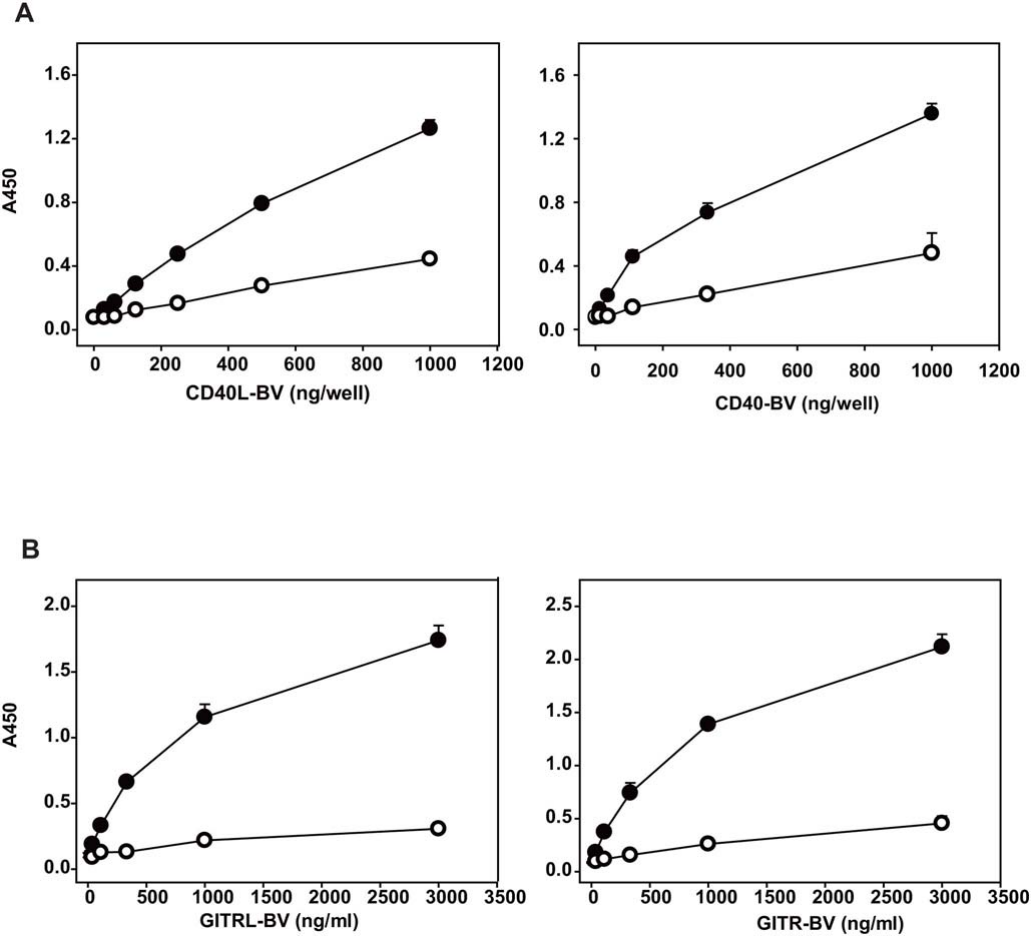
Detection of specific interaction between CD40 and CD40L or GITR and GITRL displayed on BV. (A) Left panel: binding of CD40L-BV to the immobilized CD40-BV. Wells were coated with 0.25 µg/well of CD40-BV or wild-type BV. Binding was detected by using a biotinylated anti-mouse CD40L monoclonal antibody (clone MR1) and HRP-streptavidin. Right panel: binding of CD40-BV to the immobilized CD40L-BV. Wells were coated with 1 µg/well of CD40L-BV or wild-type BV. Binding was detected by using an anti-mouse CD40 monoclonal antibody (clone 3/23) and HRP-anti rat IgG+IgM. (B) Left panel: binding of GITRL-BV to the immobilized GITR-BV. Wells were coated with 1 µg/well of GITR-BV or wild-type BV. Binding was detected by using an anti-mouse GITRL monoclonal antibody (clone YGL386) and HRP-anti rat IgG+IgM. Right panel: binding of GITR-BV to the immobilized GITRL-BV. Wells were coated with 1 µg/well of GITRL-BV or wild-type BV. Binding was detected by using an anti-mouse GITR monoclonal antibody (clone DTA-1) and HRP-anti rat IgG+IgM. Filled circles indicate binding to BV displaying respective receptors (or ligands). Open circles indicate binding to wild-type BV.

To test the application of the BV display system, we selected three representative membrane receptor-ligand pairs (CD2-CD58 [2], CD40-CD40 ligand [15], and glucocorticoid-induced TNFR family-related protein (GITR)-GITR ligand [16]), all of which are important for the interaction and activation of immune cells. We infected Sf9 cells with recombinant baculoviruses, each containing human CD2, CD58 (CD2 ligand), mouse CD40, CD40 ligand (CD40L), mouse GITR, or GITR ligand (GITRL) cDNA, and recovered the BV fractions. Immunoblot analysis using antibodies against epitope tags confirmed the expression of these membrane proteins in the BV fractions (Fig. 1). We sought to detect receptor-ligand interaction displayed on BV by ELISA system (illustrated in Fig. 2A). After CD2 (receptor)-displaying BV was immobilized in ELISA plate wells, CD58 (ligand)-displaying BV was added to the wells. We detected the binding of CD58-BV to the wells coated with CD2-BV by using a CD58-specific antibody (Fig. 2B left panel). The binding of CD58-BV was dependent on CD2 because only minimal background binding to the wells coated with wild type BV was detected (Fig. 2B left panel). Furthermore, pre-incubation of CD2-BV-coated wells with anti-CD2 antibody blocked the binding of CD58-BV (Fig. 2B right panel). Similar specific binding was observed with the opposite combination, i.e., plate-bound CD58-BV and CD2-BV in solution (Fig. 2C). Specific interactions were also detected with mouse CD40 and CD40L (Fig. 3A), and mouse GITR and GITRL (Fig. 3B). The interactions of these proteins were blocked by respective antibodies (data not shown).

### Ligand–displaying BVs bind to receptor proteins expressed on the cell surface

**Figure 4:**
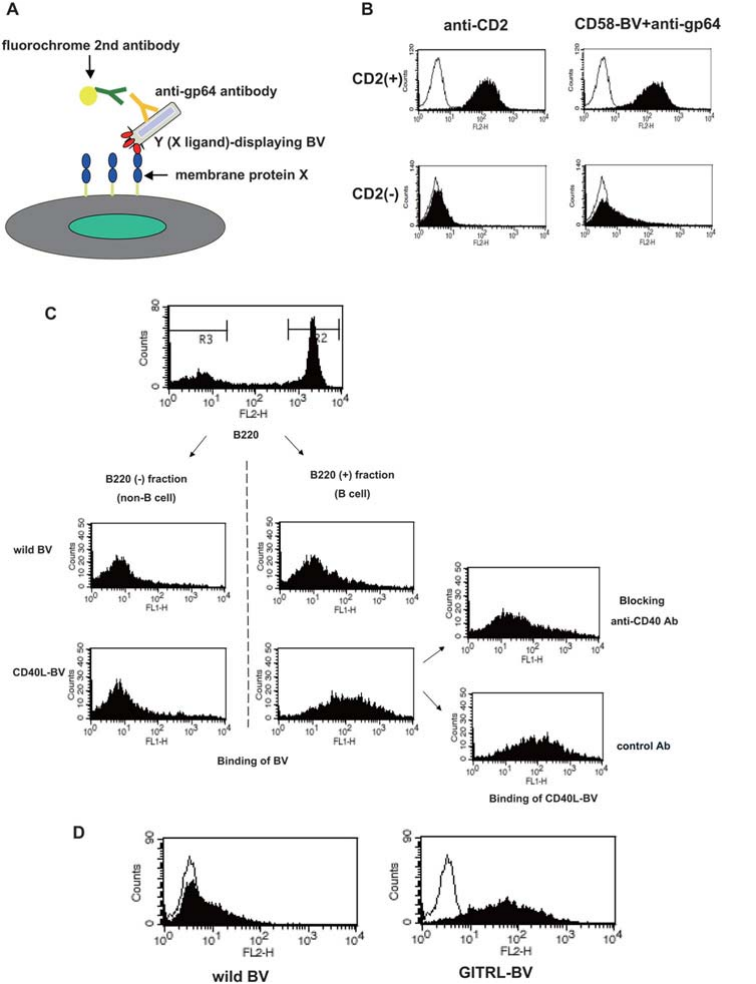
Specific binding of ligand-displaying BV to cells expressing receptor. (A) Schematic view. X corresponds to CD2 (for B), CD40 (for C), or GITR (for D). Y corresponds to CD58 (for B), CD40L (for C), or GITRL (for D). (B) CD58-displaying BV binds to CD2-positive (top), but not to CD2-negative Jurkat cells (bottom). Left: binding of an anti-CD2 monoclonal antibody (clone RPA-2.10) plus a PE-anti mouse Ig-κ chain antibody. Right: binding of CD58-displaying BV plus an anti-gp64 antibody (clone A0505A) and a PE-anti mouse Ig-κ chain antibody. The thin line indicates cells incubated with the 2nd antibody only. (C) CD40-dependent binding of CD40L-displaying BV to mouse splenic B cells. BALB/c mouse splenocytes were incubated with CD40L-displaying BV and a biotinylated anti-gp64 monoclonal antibody (clone B8147A), and then were stained with FITC-labeled streptavidin and a PE-labeled anti-B220 antibody. CD40L-BV binding to the B cells (shown in the center lower panel) was blocked by pre-incubation with an anti-mouse CD40 monoclonal antibody (clone HM40-3) (the right upper panel) but not by control hamster IgG (right lower panel). (D) Binding of GITRL-displaying BV to GITR-positive cells. GITR-expressing mouse T cell hybridoma 18.3.5 cells were incubated with GITRL-displaying BV (right) or wild-type BV (left). Binding of BV was detected with a biotinylated anti-gp64 monoclonal antibody B8147A and PE-streptavidin. The thin line indicates cells incubated without BV.

Next, we attempted to utilize the ligand protein-displaying BV as a tool to detect receptor expression on the cell surface. To this end, we detected the binding of BV to cells by flowcytometer using an antibody specific to baculoviral envelope protein gp64 and a fluorochrome-conjugated secondary antibody (illustrated in Fig. 4A). As shown in Fig. 4B, CD58-displaying BV bound to CD2-positive, but not CD2-negative Jurkat cells, a human T cell leukemia cell line. We also observed the binding of mouse CD40L-displaying BV to mouse splenic B cells, which express CD40. This binding was blocked by an anti-mouse CD40 monoclonal antibody (Fig. 4C). Furthermore, GITRL-displaying BV bound to GITR-expressing T cell hybridoma 18.3.5 cells (Fig. 4D).

### Application to expression cloning

**Figure 5:**
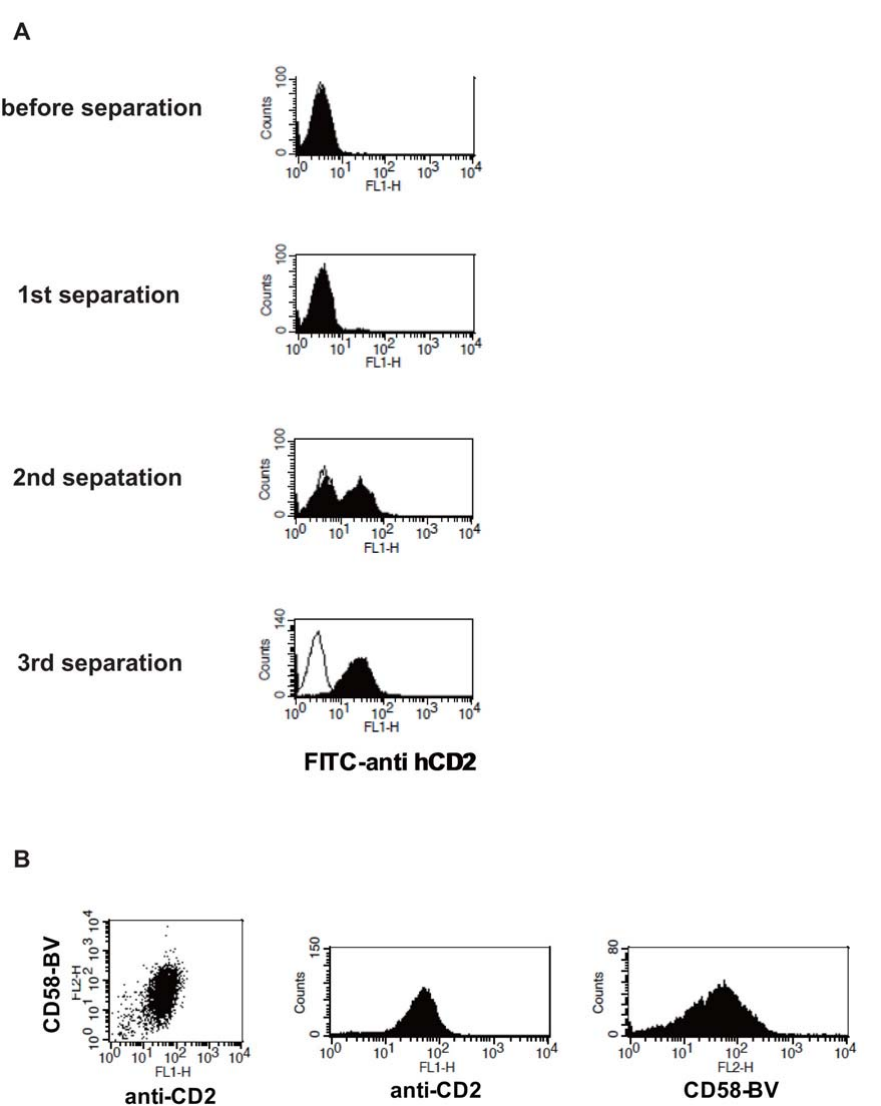
Expression cloning of human CD2 by using CD58-displaying BV as the probe. (A) Enrichment of human CD2-positive cells from BaF/3 cells transfected with a human T cell cDNA library. The staining of cells with an FITC-labeled anti humanCD2 monoclonal antibody is shown. The thin line indicates cells incubated without anti-CD2 antibody. (B) Binding of anti-human CD2 antibody and CD58-displaying BV to BaF/3 cells isolated by magnetic sorting with CD58-BV. After the 3rd magnetic sorting and subcloning, cells were stained with an FITC-labeled anti-human CD2 monoclonal antibody and CD58-BV plus biotinylated anti-gp64 antibody and PE-streptavidin. Staining of a representative of three clones is shown.

Our results prompted us to explore the possibility of utilizing BV displaying membrane proteins as the probe for expression cloning of receptor (or ligand) cDNA. To this end, we transfected BaF/3 cells with a human T cell cDNA library using a retroviral transduction system. Cells bound to CD58-displaying BV were selected with an anti-viral gp64 antibody plus secondary antibody-coated magnetic beads. After magnetic sorting of the BV-bound cells three times, cells expressing human CD2 were enriched (Fig. 5A). The recovered cells were cloned by limiting dilution. PCR of cloned BaF/3 cell genomic DNA with retroviral vector-derived primers amplified the human CD2 cDNA sequence. Furthermore, flowcytometric analysis confirmed that these clones expressed human CD2 and that CD58-BV bound to these cells (Fig. 5B).

## Discussion

Functional membrane proteins such as cell surface receptors, transporters, or enzymes, have been shown to heterologously express on BV [9]–[14, reviewed in 9]. In this study, we further applied this BV display system in an effort to detect interactions between membrane receptors and ligands in immune cells. We infected Sf9 cells with recombinant baculoviruses, individually encoding CD2, CD58, CD40, CD40L, GITR or GITRL, and recovered the BV fractions expressing these membrane proteins. We observed specific binding of CD58 (ligand)-displaying BV to CD2 (receptor)-displaying BV by ELISA (Fig. 2). Interactions were also detected between CD40 and CD40L, and between GITR and GITRL by this method (Fig. 3). We also demonstrated that membrane protein-displaying BV can be utilized to detect cells expressing their specific receptors (Fig. 4). Furthermore, we successfully cloned CD2 cDNA from an expression cDNA library by using CD58 (CD2 ligand)-displaying BV as the probe (Fig. 5).

The BV display system described here is expected to have an advantage over other systems currently in use. It should be applicable to various membrane receptor proteins and their ligands (or counter receptors), which are also membrane proteins. Since membrane proteins displayed on BV have the capacity to move laterally on the surface of the viral membrane, they are able to form oligomers. Currently, the fusion of the extracellular domain of membrane proteins with the Fc portion of immunoglobulin (Fc-fusion protein) is commonly used as a technique to reconstitute dimer or pentamer structures and to detect receptors (or ligands) [3], [4], [17]. However, it is difficult to reconstitute functional hetero-oligomeric complexes by this method. Furthermore, some proteins may not retain the proper conformation after such manipulation. Since heterologous membrane proteins express on BV in their native forms, it is highly likely that the BV display system reconstitutes functional hetero-oligomeric protein complexes. The low background of the intrinsic expression of these immune receptors or ligands in BV also provides a critically important advantage.

Recently several investigators have reported that they were able to attach retroviral or adenoviral particles to optical biosensor surfaces and then detect interaction with antibodies or ligands specific to the receptors displayed on the virus [18], [19]. Baculoviral particles can also be immobilized on a solid surface, and proteins displayed on BV retain the ability to bind their ligands (or receptors) for at least several months at 4°C. The potential application to the development of a biochip sensor is another advantage of the BV display system.

In summary, we have developed a new method for detecting membrane protein interactions using the BV display system. The BV display system reported here is expected to be highly useful for the analysis of cell-to-cell interaction mechanisms important in the immune system, neural system, or developmental organ formation. It also can be applied to develop screening systems for therapeutic antibodies and/or compounds.

## Materials and Methods

### Antibodies and reagents

The horseradish peroxidase (HRP)-conjugated monoclonal mouse anti-FLAG M2 antibody was from Sigma, and the monoclonal mouse anti-HA antibody (F-7) was from Santa Cruz. HRP-conjugated goat anti-mouse IgG, and anti-rat IgM+IgG were from Jackson ImmunoResearch Laboratories and Southern Biotechnology, respectively. Monoclonal mouse anti-human CD2 (clone RPA-2.10, unconjugated or biotinylated), anti-human CD58 (clone 1C3 (AICD58.6)), rat anti-mouse CD40 (clone 3/23), hamster anti-mouse CD40 (clone HM40-3), and biotinylated hamster anti-mouse CD40L (clone MR1) antibodies were from BD PharMingen. Unconjugated monoclonal hamster anti-mouse CD40L (clone MR1) and FITC or PE-conjugated monoclonal rat anti-mouse immunoglobulin κ chain (clone H139.52.1) antibodies were from Beckman Coulter. A monoclonal rat anti-mouse GITR antibody (clone DTA-1) was generated as previously described [20]. A monoclonal rat anti-mouse GITRL antibody (clone YGL386) was from Serotec. Biotinylated mouse anti-human CD58 (clone TS2/9.1.4.3) and rat anti-mouse CD40 (clone 3/23) were from Ancell Corp. and Caltag Laboratories, respectively. HRP-conjugated streptavidin was from Vector Laboratories. FITC or PE-conjugated streptavidin was from BD PharMingen. Monoclonal mouse antibodies against baculoviral envelope protein gp64 (clones A0505A and B8147A) were established in our laboratory.

### Recombinant baculovirus construction and Sf9 cell culture

The cDNAs for human CD2, CD58, and mouse CD40 were amplified by PCR from human lymph node and mouse spleen cDNA libraries (TAKARA Bio), respectively. The cDNA for mouse CD40L was purchased from ATCC. The cDNA for mouse GITR was amplified by RT-PCR from poly A-positive RNA isolated from an 18.3.5 T cell hybridoma, which expresses GITR [20]. The cDNA for mouse GITRL was cloned in our laboratory. The FLAG tag sequence was added to the 3′-terminus of CD2 and 5′-terminus of CD40L and GITRL cDNAs. The sequence for the HA tag was added to the 3′-terminus of CD58, CD40, and GITR cDNAs. The nucleotide sequences of these DNA constructs were confirmed. The DNA fragments were subcloned into the baculoviral transfer vector pBlueBac4.5 (Invitrogen.). Recombinant baculoviruses were generated using a Bac-N-Blue system (Invitrogen) according to the manufacturers' instructions. Sf9 cells were cultured as described [11].

### Preparation of the budded baculovirus (BV) fractions

Sf9 cells (2×10^6^ cells/ml) were infected with recombinant baculovirus at the multiplicity of infection (M.O.I.) of 5. Seventy-two hours after infection, the BV fraction was isolated from the culture supernatant of infected Sf9 cells as described [10], [11]. The pellets of the BV fraction were resuspended in Tris-buffered saline (TBS) containing 1 mM EDTA, 50 µM E64, 2 µg/ml aprotinin, and 10 µg/ml leupeptin, and stored at 4°C. The expression of recombinant proteins in BV fraction was confirmed by SDS- polyacrylamide gel electrophoresis and Western blot analysis using an HRP-anti FLAG M2 antibody or an anti-HA antibody (F-7) plus HRP-goat anti mouse IgG. Samples were not heat-treated so as to minimize aggregation.

### Enzyme-linked immunosorbent assay (ELISA)

The BV displaying human CD2 was diluted with TBS and adsorbed to the wells of a 96-well ELISA plate (Greiner Bio-One) overnight at 4°C. Wells were washed with phosphate-buffered saline (PBS) and blocked with TBS containing 40% BlockAce (Dainippon Sumitomo Pharma) for 1 h at room temperature. The BV displaying human CD58 was diluted with Hanks' Balanced Salt Solution (HBSS) containing 40% BlockAce, added to the wells, and incubated for 1 h at room temperature. Wells were washed with PBS, and incubated with a mouse monoclonal antibody specific to human CD58 (clone 1C3 (AICD58.6)). After being washed with PBS containing 0.01% Tween 20, wells were incubated with HRP-conjugated goat anti-mouse IgG, washed, and further reacted with the tetramethylbenzidine liquid substrate (Sigma). The reaction was terminated by the addition of 0.5 M H_2_SO_4_, and absorbance at 450 nm was quantitated using a 96-well plate reader. Similar assays were carried out with the combination of human CD58-BV bound to the plate and CD2-BV in the solution. Binding between the BVs individually displaying mouse CD40 and CD40L, or GITR and GITRL was also detected by using a similar ELISA method.

For the blocking experiment, CD2-BV was immobilized to the ELISA plate and wells were blocked with BlockAce, as described above. After pre-incubating wells with a monoclonal anti-human CD2 antibody (clone RPA-2.10) for 15 min at room temperature, CD58-BV was added to the wells. Wells were incubated for 1 h at room temperature, washed, and reacted with a biotinylated anti-CD58 antibody (clone TS2/9.1.4.3), then, with HRP-conjugated streptavidin. Similar blocking assays were carried out with the combination of human CD58-BV bound to the plate and the CD2-BV in the solution using anti-human CD58 (clone 1C3 (AICD58.6)) and biotinylated anti-human CD2 (clone RPA-2.10) for the blocking and detection antibodies, respectively. Binding between the plate-bound mouse CD40-BV and mouse CD40L-BV in the liquid phase was also blocked by an anti-mouse CD40 monoclonal antibody (clone HM40-3). A biotinylated anti-mouse CD40L monoclonal antibody (clone MR1) was used as the detection antibody. Similar blocking assays were carried out with a combination of mouse CD40L-BV bound to the solid phase and CD40-BV in the liquid phase by using anti-mouse CD40L (clone MR1) and biotinylated anti-mouse CD40 (clone 3/23) for the blocking and detection antibodies respectively.

### Flowcytometric analysis

Cells were washed with FACS buffer (PBS containing 1% fetal calf serum (FCS)) and resuspended in Dulbecco's Modified Eagle's Medium (DMEM) containing 20% FCS at approximately 5×10^6^ cells/ml. One hundred microliters of cell suspension were incubated with BV for 30 min at 4°C, washed twice with FACS buffer, and incubated with an anti-baculoviral envelope protein gp64 monoclonal antibody (clone A0505A or B8147A) for 30 min on ice. The cells were washed, and then further incubated with FITC or PE-labeled rat anti-mouse Ig-κ monoclonal antibody for 30 min on ice. The cells were washed again, resuspended in FACS buffer, and analyzed on a FACS Calibur flowcytometer (Becton Dickinson).

### Expression cloning

A human T lymphocyte cDNA library was constructed as described [21] by using the pBabeX retroviral vector. Plat-E packaging cells [22] were transfected with the cDNA library using FuGene 6 (Roche Diagnostics). Two days later, culture supernatant containing retroviruses was collected and used to infect 2.4×10^6^ cells of mouse pro-B cell line BaF/3 (purchased from RIKEN BRC Cell Bank) in the presence of polybrene (8 µg/ml). After 4 h, fresh media, twice as much volume as that of the viral stock, was added and cells were cultured overnight. On the next day, cells were washed, resuspended in the fresh media, and cultured overnight once again. Two days after the day of infection, 8×10^7^ cells were washed with DMEM containing 20% FCS and incubated with 80 µg of hCD58-BV for 30 min at 4°C, washed again, then incubated with 10 µg of A0505A anti-baculoviral gp64 monoclonal antibody for 20 min at 4°C. Cells were further incubated with magnetic microbeads coated with goat anti-mouse IgG (Miltenyl Biotech). Cells bound with hCD58-BV were isolated by magnetic cell sorting using a MACS system (Miltenyl Biotech). After three time purification by magnetic sorting, isolated cells were cloned with limiting dilution. The cDNAs derived from the retroviral library were amplified from the genomic DNA of individual clones by PCR using upstream and downstream retroviral vector primers (5′-CAGCCCTCACTCCTTCTC-3′ and 5′- CCCTTTTTCTGGAGACTAAAT-3′).
